# Performance of two rapid diagnostic tests for malaria diagnosis at the China-Myanmar border area

**DOI:** 10.1186/1475-2875-12-73

**Published:** 2013-02-22

**Authors:** Juan Yan, Nana Li, Xu Wei, Peipei Li, Zhenjun Zhao, Lili Wang, Siying Li, Xiaomei Li, Ying Wang, Shuying Li, Zhaoqing Yang, Bin Zheng, Guofa Zhou, Guiyun Yan, Liwang Cui, Yaming Cao, Qi Fan

**Affiliations:** 1 Department of Immunology, College of Basic Medical Sciences, China Medical University, Shenyang, Liaoning, China; 2 Department of Parasitology, Kunming Medical University, Kunming, Yunnan, China; 3 Dalian Institute of Biotechnology, Dalian, Liaoning, China; 4 Third Military Medical University, Chongqing, China; 5 Institute of Parasitic Diseases, Shanghai, China; 6 University of California, Irvine, CA, USA; 7 Department of Entomology, Pennsylvania State University, University Park, PA, USA

**Keywords:** Rapid diagnostic tests (RDTs), Malaria diagnosis, Microscopy, PCR, Sensitivity, Specificity

## Abstract

**Background:**

Rapid diagnostic tests (RDTs) have become an essential tool in the contemporary malaria control and management programmes in the world. This study aims to evaluate the performance of two commonly used RDTs for malaria diagnosis in the China-Myanmar border area.

**Methods:**

A total 606 febrile patients in the China-Myanmar border were recruited to this study and were diagnosed for malaria infections by microscopy, two RDTs tests (Pf/Pan device, and Pv/Pf device) and nested PCR.

**Results:**

Malaria parasites were found in 143 patients by microscopy, of which 51, 73, and 19 were *Plasmodium falciparum*, *Plasmodium vivax* and *P. falciparum*/*P. vivax* mixed infections, respectively. Compared to microscopy, the sensitivity of the Pf/Pan device was 88.6% for *P. falciparum* and 69.9% for *P. vivax* with the specificity of 90.4%. For a subset of 350 patients, the sensitivity of the Pf/Pan device and Pv/Pf device for detection of *P. falciparum* was 87.5% and 91.7%, respectively; and for detection of *P. vivax* was 72.0% and 73.8%, respectively. The specificity of the Pf/Pan device and Pv/Pf device was 94.3% and 96.5%, respectively. Nested PCR detected malaria parasites in 174 of 606 samples, of which 67, 79, two and 26 were *P. falciparum*, *P. vivax*, *P. ovale* and *P. falciparum*/*P. vivax* mixed infections, respectively. Compared to nested PCR, all other methods had sensitivity below 80%, suggesting that a significant number of cases were missed.

**Conclusions:**

Compared to PCR, both microscopy and RDTs had lower sensitivities. RDTs had similar performance to microscopy for *P. falciparum* diagnosis, but performed worse for *P. vivax* diagnosis. Other RDT products should be selected with higher sensitivity (and good specificity) for both *P. falciparum* and *P. vivax* diagnosis.

## Background

Malaria is a highly prevalent disease in tropical and subtropical regions, affecting half of the world’s population in 108 countries, resulting in almost one million deaths annually [1]. Malaria in human can be caused by one of five malaria parasites (*Plasmodium falciparum*, *Plasmodium vivax*, *Plasmodium malariae*, *Plasmodium ovale* and *Plasmodium knowlesi*), which have different geographical distributions. *Plasmodium falciparum* causes the most severe form of the disease and tends to predominate in tropical areas. *Plasmodium vivax* is the predominant species outside Africa. In recent years, it has been increasingly recognized that *P. vivax* is also associated with severe symptoms [2], which changed the traditional view of this malaria as “benign tertian”. The great impact of the 130–435 million *P. vivax* infections each year and the significant lag in research and prevention justifies considering *P. vivax* malaria as a neglected tropical disease [3,4]. In many parts of the world, such as Southeast Asia, *P. vivax* occurs sympatrically with *P. falciparum*. Since these two parasites require different drug treatments, accurate diagnosis is required to differentiate these two species in areas of co-existence.

Microscopic examination of Giemsa-stained blood smears under a light microscope remains the gold standard method for malaria diagnosis. However, this technique requires a relatively long observation time and well-trained microscopists. Misdiagnosis often happens in samples with low parasitaemia, especially when drugs are taken inappropriately [5,6]. Furthermore, microscopic diagnosis of *P. vivax* is more challenging, because parasite density during *P. vivax* infections is often low. Recently, the use of molecular methods such as PCR, for the diagnosis of malaria has proved to be highly sensitive and specific [7,8], but drawbacks such as a requirement for equipment, higher cost, and a lengthy procedure limit their routine [9-11]. Malaria rapid diagnostic tests (RDTs) have become very popular in various endemic settings [12], especially in areas where microscopic expertise is lacking. They are now an essential tool in malaria management during the malaria elimination/eradication campaign [13]. However, the wide variety of RDTs and their different performance under different endemic settings suggest that careful comparison of RDTs is needed before mass deployment for diagnosis.

RDTs are designed using antibodies against parasite species-specific or genus-specific antigens, such as *P. falciparum*-specific histidine-rich protein-2 (PfHRP2) and parasite lactate dehydrogenase (pLDH). However, the performance of RDTs is easily affected by humidity and extreme temperatures. In addition, persistence of antigens that may remain in the circulation of a patient after treatment may give false positive results [14]. In malaria endemic areas of the Greater Mekong Subregion (GMS), the four human malaria species often co-exist, but with *P. falciparum* and *P. vivax* being the predominant species. In this region, cases of human infected by the monkey malaria parasite *P. knowlesi* were also reported [15,16]. Several types of RDTs have been evaluated in these areas, and most of them had poor performance for low levels of parasitaemia (e.g., <500 parasites/μL), which is especially true for vivax malaria [17,18]. In recent years, with extensive supports from the Global Fund to fight AIDS, Tuberculosis and Malaria, the malaria control programme at the China-Myanmar border area has been substantially strengthened. Among the control measures is the mass use of RDTs for malaria diagnosis. Specifically, two types of RDTs have been extensively used based on co-existence of *P. falciparum* and *P. vivax* in this area: a Pf/Pan test device and a Pv/Pf test device. However, their performance for malaria diagnosis under this specific endemic setting has not been evaluated. In this study, the performance of the two RDTs was compared with that of microscopy and PCR for the diagnosis of *P. falciparum* and *P. vivax* malaria.

## Methods

### Study site and patients

The study was conducted in 2011 in Laiza township area along the China-Myanmar border. Malaria in this region, caused mainly by *P. falciparum* and *P. vivax* infections, is perennial with distinct seasonality, which occurs mostly in the rainy season from April to November. A total of 606 patients attending local malaria clinics and hospital were recruited into the study based on the following criteria: suspected of uncomplicated malaria, having fever with axillary temperature above 37.5°C at the time of examination and willing to participate in the study. The study population has a sex ratio of ~1. Their ages ranged from six months to 88 years with a median of 20.3 years. The relative proportions of patients under 15 years, between 16 and 50 years, and >50 years were 52.9, 39.3, and 7.8%, respectively. The study protocol was reviewed and approved by the Institutional Review Board of Kunming Medical University. All participants or legal guardians gave written informed consent before entering the study. Finger-prick blood was obtained for blood films, RDTs and PCR.

### Microscopic examinations

Thick and thin blood films were prepared from peripheral blood. The slides were stained with Giemsa and screened for the presence of parasites and identification of parasite species. Stained blood films were examined with (a 100×) oil immersion lens. Parasite density was determined by counting the parasites and leucocytes, assuming 8,000 leucocytes/μL of blood [19]. Blood films were examined by an experienced microscopist who was ‘blinded’ to the results of additional diagnostic tests. Smears were considered negative if no parasite was seen in 100 oil immersion fields on a thick blood film. All the slides were double checked blindly by a second, independent microscopist and the results were combined. Parasite density was calculated by determining the number of parasites per 200 white blood cells in a thick blood film.

### RDTs for malaria

The RDTs used in this study are One Step Malaria Pf/Pan test (Wondfo, China) and Malaria Pv/Pf test device (Cat. No. 200317, Tycolpharm Co., Limited, UK). The major target antigens of the Pf/Pan test device are PfHRP2 and pan-pLDH, which are specific for *P. falciparum* and all human *Plasmodium* species, respectively. The Pv/Pf test device is based on PfHRP2 antigen and Pv*-*pLDH antigen, which are specific for *P. falciparum* and *P. vivax*, respectively. Five microlitres of fresh whole blood was added to the card pad, and three drops of specific lying agent were added. The RDT result was read in 15–20 min according to the manufacturer’s instructions and immediately recorded. The test was considered valid when the control line on the immune-chromatographic test strip was shown. For the Pf/Pan test device, it was counted as *P. falciparum-*positive if the line detecting *P. falciparum*-specific PfHRP2 was positive or if the lines detecting both PfHRP2 and pan-pLDH were positive; as non-falciparum if only the pan-pLDH line was positive. For the Pv/Pf test device, it was counted as *P. falciparum*- or *P. vivax*-positive if one of both specific test lines were positive. The readers of RDTs were blinded to the results of microscopy and PCR. All 606 cases in this study were diagnosed by the Malaria Pf/Pan test device. Later, the Malaria Pv/Pf test device was added as a comparison and 350 cases were diagnosed by both RDTs.

### Diagnosis by PCR

Fresh blood samples were spotted on Whatman 3 paper, air-dried at room temperature, and stored individually in a zipper plastic bag at −20°C until use to prevent DNA degradation. Genomic DNA was extracted from dried blood spots using QIAamp DNA Micro Kit (Qiagen) according to the manufacturer’s instruction. Nested PCR for *Plasmodium* species was slightly modified based on previously published work using the small subunit (SSU ) rRNA gene [20,21]. *Plasmodium* genus-specific primers were shown in Table 1. For the primary PCR reactions, 2 μL of genomic DNA were used in a 25 μL reaction with outer primers rPLU5 and rPLU6, and 30 cycles (94°C for 1 min, 55°C for 2 min and 72°C for 2 min) were performed. For nested PCR, 2 μL of the primary PCR product were used as the template with species-specific primers for the four human malaria species and *P. knowlesi* in separate reaction tubes. After another 30 cycles of amplification (94°C for 40 s, 58°C for 1 min and 72°C for 2 min), the PCR products were separated in 1% and 2% agarose gels for primary and nested PCR, respectively. DNA bands were stained with ethidium bromide and visualized under a UV light. The primers and expected sizes of the PCR fragments of the SSU rRNA genes are shown in Table 1.

**Table 1:** Primers based on the 18S rRNA gene in malaria parasites for nested PCR diagnosis of malaria infections

**Species**	**Primer**	**Sequence (5’-3’)**	**Size of PCR product (bp)**
*Plasmodium sp*.	rPLU5	CCTGTTGTTGCCTTAAACTTC	1100
	rPLU6	TTAAAATTGTTGCAGTTAAAACG	
*P. falciparum*	rFAL1	TTAAACTGGTTTGGGAAAACCAAATATATT	205
	rFAL2	ACACAATGAACTCAATCATGACTACCCGTC	
*P. vivax*	PV18SF	GAATTTTCTCTTCGGAGTTTATTC	419
	PV18SR	GTAGAAAAGGGAAAGGGAAACTGTTA	
*P. malariae*	PM18SF	GAGACATTCATATATATGAGTGTTTCT	423
	PM18SR	GGGAAAAGAACGTTTTTATTAAAAAAAAC	
*P. ovale*	PO18SF	GAAAATTCCTTTTGGAAATTTCTTAG	410
	PO18SR	GGGAAAAGGACACTATAATGTATC	
*P. knowlesi*	PK18SF	GAGTTTTTCTTTTCTCTCCGGAG	424
	PK18SR	GGGAAAGGAATCACATTTAACGT	

### Statistical analysis

The RDT results were compared with those from microscopy and nested PCR. Sensitivity and specificity were calculated with 95% confidence intervals (C.I.) in two separate analyses: (1) diagnostic performance of RDTs in comparison with the microscopic method, and (2) comparative diagnostic performances of RDTs in comparison with the microscopy and PCR as the references. Based on the microscopic results, the RDT results were considered true positive (TP), true negative (TN), false positive (FP), and false negative (FN) using the interpretation criteria presented in Table 2. Sensitivity and specificity were calculated as TP/(TP+FN) and TN/(TN+FP), respectively. Statistical analysis was performed by the Chi-square test using the SPSS version 15.0 and the significance level was set as < 0.05.

**Table 2:** Interpretation of the results for the Pf/Pan RDT

**For *P. falciparum***		
Test Line(s) visible	Species identification by microscopy (or corrected by PCR)	
	*P. falciparum* or as a mixed infection with *P. falciparum*	*P. vivax, P. ovale, P. malariae, P. knowlesi* /no parasites detected
Only Pf or both Pf and Pan line visible	True positive (TP)	Species mismatch**/false positive (FP)
No test line or only Pan line visible	False negative/species mismatch* (FN)	True Negative (TN)
**For the non-*falciparum *species**		
Test Line(s) visible	Species identification by microscopy (or corrected by PCR)	
	*P. vivax, P. ovale, or P. malariae*	*P. falciparum*/no parasites detected
only Pan line visible	True positive (TP)	Species mismatch*/false positive (FP)
No test line or Only Pf or both Pf and Pan line visible	False negative/species mismatch**(FN)	True Negative(TN)

* *P. falciparum* or as a mixed infection with *P. falciparum* diagnosed as non-*falciparum* species.

** Non-*falciparum* species diagnosed as *P. falciparum* or as a mixed infection with *P. falciparum*.

## Results

Blood samples were collected from a total of 606 patients. All samples were evaluated by the Pf/Pan test device, while a subset of 350 were also evaluated by the Pv/Pf test device. Of the 606 samples, malaria parasites were found in 143 by microscopy; 51, 73, and 19 were *P. falciparum*, *P. vivax* and *P. falciparum*/*P. vivax* mixed infections, respectively (Table 3). No other malaria parasite species were detected by microscopy. The parasite density ranges of *P. falciparum* were 40–105,920 parasites/μL. For *P. vivax,* the majority of cases had parasite density ranging from 80 to 17,800 parasites/μL. One *P. vivax* case had a deviant parasite density of >200,000 parasites/μL based on leucocyte number probably due to a low leucocyte count in this patient. Because *P. falciparum* single infections and mixed species infections containing *P. falciparum* cannot be differentiated by the Pf/Pan device, the microscopy results were grouped into “*P. falciparum* and mixed infections with *P. falciparum*” and “Non-falciparum”. Based on this interpretation, the Pf/Pan device detected 33 single *P. falciparum* infections (only the PfHRP2 line visible), 56 non-falciparum cases (only the pan-pLDH line visible) and 70 *P. falciparum* infections/potentially mixed infections (both lines visible) (Table 3). Compared with the results by microscopy, the Pf/Pan device detected 62 and 51 samples as true positives for *P. falciparum* and non-falciparum, respectively. From this result, the sensitivity of Pf/Pan device was 88.6% for *P. falciparum* and 69.9% for *P. vivax* with the specificity of 90.4% (Table 4).

**Table 3:** Detection results of malaria infections by the Malaria Pf/Pan test in comparison with microscopy (N=606)

**Method**	**Species**	**Species identified by microscopy**				**Total**
		***P. falciparum***	***P. vivax***	***P. falciparum + P. vivax***	***Negative***	
Pf/Pan Test	*P. falciparum*	18	3	2	10	33
	*Pan* (non-*falciparum*)	1	51	1	3	56
	*P. falciparum + Pan*	27	13	15	15	70*
	*Negative*	5	6	1	435	447
	Total	51	73	19	463	606

* For the Pf/Pan device, these cases had both test lines visible, suggesting they were either *P. falciparum* single infections or *P. falciparum*/non-*falciparum* mixed infections.

**Table 4:** **Performance of two RDTs for detection of *P. falciparum *and *P. vivax *with microscopy as the gold standard**

	**Pf/Pan (N=606)**	**Pf/Pan (N=350)**	**Pv/Pf (N=350)**
Sensitivity for *P. falciparum*	88.6 (85.9-91.3)	87.5 (83.8-91.2)	91.7 (88.5-94.8)
Sensitivity for *P. vivax* (non-falciparum for Pf/Pan)	69.9 (66.0-73.8)	72.0 (66.9-77.1)	73.8 (68.7-78.8)
Specificity	90.4 (87.8-93.1)	94.3 (91.4-97.1)	96.5 (94.3-98.7)

Data are presented as percentage (95% confidence interval; CI).

For the subset of 350 samples, microscopy detected 37 samples containing *P. falciparum*, 50 samples containing *P. vivax* and 11 were diagnosed as mixed species infections (Table 5). Since the Pv/Pf device is able to differentiate *P. falciparum* and *P. vivax* infections, the microscopy results were grouped into “infections containing *P. falciparum”* and “infections containing *P. vivax*”. The Pv/Pf device detected 40 single *P. falciparum* infections, 42 *P. vivax* infections, and 8 mixed species infections (Table 5). Compared to microscopy, the Pv/Pf device detected 44 and 45 samples as true positives for *P. falciparum* and *P. vivax*, respectively, whereas the Pf/Pan device detected 42 and 36 samples as true positives for *P. falciparum* and *P. vivax*, respectively. The sensitivity of Pf/Pan device and Pv/Pf device for detection of *P. falciparum* was 87.5% and 91.7%, respectively; and for detection of *P. vivax* was 72.0% and 73.8%, respectively (Table 4). The specificity of Pf/Pan device and Pv/Pf device was 94.3% and 96.5%, respectively (Table 4).

**Table 5:** Detection results of malaria infections by Malaria Pv/Pf test and Malaria Pf/Pan test in comparison with the microscopic method (N=350)

**Method**	**Species**	**Species identified by microscopy**					**Total**
		***P. falciparum***	***P. vivax***	***P. falciparum + P. vivax***	***P. ovale***	***Negative***	
Pv/Pf Test	*P. falciparum*	32	1	6	0	1	40
	*P. vivax*	0	39	0	0	3	42
	*P. falciparum + P. vivax*	2	2	4	0	0	8
	*Negative*	3	8	1	0	248	260
Pf/Pan Test	*P. falciparum*	17	1	2	0	2	22
	*Pan*(non-*falciparum*)	0	36	0	0	2	38
	*P. falciparum + Pan*	15	8	8	0	2	33*
	*Negative*	5	5	1	0	246	257
	Total	37	50	11	0	252	350

* For the Pf/Pan device, these cases had both test lines visible, suggesting they were either *P. falciparum* single infections or *P. falciparum*/non-*falciparum* mixed infections.

It has been observed that some RDTs do not perform well when the parasite density is below 500 parasites/μL. For *P. falciparum,* both RDTs had significantly higher sensitivity for cases with a parasite density above 500 parasites/μL (>92%) than those with a density below 500 parasites/μL (<75.0%) (Table 6). However, for *P. vivax* malaria, regardless of the parasite density, both RDTs displayed sensitivity of below 79%. Both RDTs had similar detection limits; they were >240 and >320 parasites/μL for *P. falciparum* and *P. vivax,* respectively.

**Table 6:** **Comparative sensitivity of the two RDTs for detection of *P. falciparum *and *P. vivax *in comparison with the microscopic method, categorized according to parasite density**

	**Density (Parasites/μL)**	**Pf/Pan (N=606)**		**Pf/Pan (N=350)**		**Pv/Pf (N=350)**	
		**TP (N)**	**Sensitivity (%)**	**TP (N)**	**Sensitivity (%)**	**TP (N)**	**Sensitivity (%)**
*P. falciparum*	>500	51	92.7 (86.0-99.4)	34	94.4 (87.3-100)	35	97.2 (92.1-100)
	<500	11	73.3 (69.4-77.3)	8	66.7 (61.0-72.4)	9	75.0 (70.1-80.2)
*P. vivax* (or non-falciparum)	>500	40	69.0 (56.7-81.2)	28	71.8 (57.1-86.5)	36	78.3 (63.2-93.2)
	<500	11	73.3 (69.4-77.3)	8	72.7 (67.4-78.1)	9	60.0 (54.0-66.0)

Sensitivity is presented as percentage (95% confidence interval; CI). *TP* true positive.

**Figure 1:**
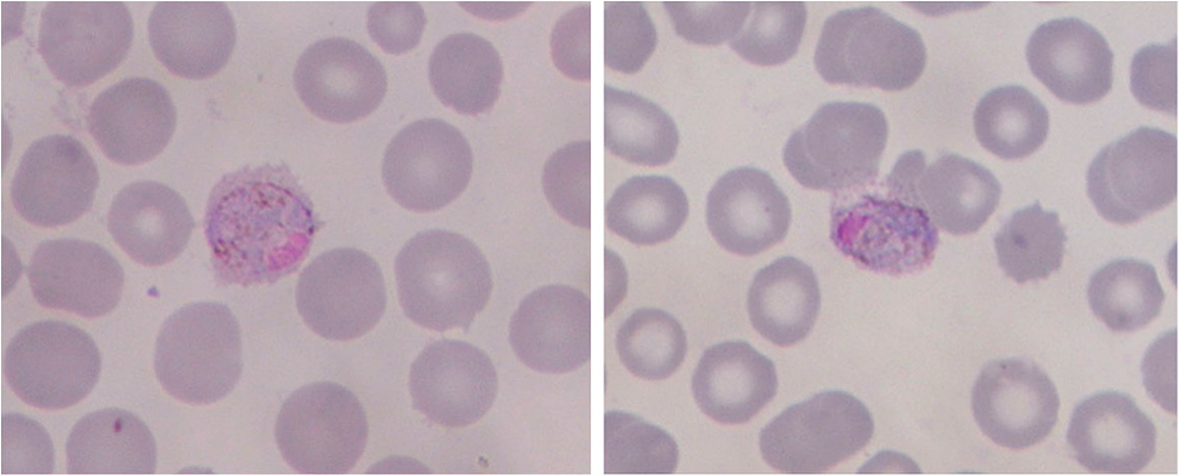
**Blood smears showing parasitized erythrocytes by *P. ovale *in two malaria patients.** The two patients with febrile illness attended a malaria clinic at the China-Myanmar border and were diagnosed for malaria. Both cases were only detected by PCR but missed by microscopy and RDTs.

To further evaluate the performance of these two RDTs, all 606 samples were examined by nested PCR. Nested PCR detected malaria parasites in 174 samples, of which 67, 79, two and 26 were *P. falciparum*, *P. vivax*, *P. ovale* and *P. falciparum*/*P. vivax* mixed infections, respectively. No *P. knowlesi* infections were detected by PCR (Table 7). For the subset of 350 samples, 113 samples were identified by nested PCR as infection with malaria parasites. Of which, 40, 52, and 21 were *P. falciparum*, *P. vivax*, and *P. falciparum*/*P. vivax* mixed infections, respectively (Table 8). Among all detection methods, PCR was the most sensitive one. If PCR was used as the gold standard, the sensitivity of microscopy for *P. falciparum* and *P. vivax* was 71.0% and 73.3% in 606 detected samples, and 75.4% and 74.0% in the 350 subset samples, respectively (Table 9). The sensitivity of Pf/Pan test for *P. falciparum* and *P. vivax* was 81.7% and 64.6% in 606 detected samples, and 77.1% and 63.5% in the 350 subset samples, respectively (Table 9). For the subset of 350 samples analyzed by Pv/Pf devices, the sensitivity was 72.1% for *P. falciparum* and 58.9% for *P. vivax,* respectively (Table 9). Microscopy and the two RDTs had similar levels of specificity ranging from 94.7% to 96.3% (Table 9). It is noteworthy that although the Pf/Pan device is designed to identify all human malaria parasite species, the two *P. ovale* infections identified by PCR were missed by this device and by microscopy, possibly due to the low parasitaemia in the *P. ovale* infections (containing 120 parasites/μL and 320 parasites/μL, respectively). Re-examination of the *P. ovale* slides by microscopy did detect *P. ovale*-parasitized erythrocytes (Figure 1).

**Table 7:** Detection results of malaria infections by microscopy and Malaria Pf/Pan test in comparison with the Nested PCR (N=606)

**Method**	**Species**	**Species identified by nested PCR**					**Total**
		***P. falciparum***	***P. vivax***	***P. falciparum + P. vivax***	***P. ovale***	***Negative***	
Microscopy	*P. falciparum*	39	2	9	0	1	51
	*P. vivax*	1	61	9	0	2	73
	*P. falciparum + P. vivax*	12	1	6	0	0	19
	*P. ovale*	0	0	0	0	0	0
	*Negative*	15	15	2	2	429	463
Pf/Pan Test	*P. falciparum*	16	1	7	1	8	33
	*Pan*(non-*falciparum*)	0	51	5	0	0	56
	*P. falciparum + Pan*	40	10	13	0	7	70*
	*Negative*	11	17	1	1	417	447
	Total	67	79	26	2	432	606

* For the Pf/Pan device, these cases had both test lines visible, suggesting they were either *P. falciparum* single infections or *P. falciparum*/non-*falciparum* mixed infections.

**Table 8:** Detection results of malaria infections by microscopy, Malaria Pf/Pan test and Malaria Pv/Pf test in comparison with the Nested PCR (N=350)

**Method**	**Species**	**Species identified by Nested PCR**					**Total**
		***P. falciparum***	***P. vivax***	***P. falciparum + P. vivax***	***P. ovale***	***Negative***	
Microscopy	*P. falciparum*	29	1	6	0	1	37
	*P. vivax*	0	40	9	0	1	50
	*P. falciparum + P. vivax*	6	0	5	0	0	11
	*P. ovale*	0	0	0	0	0	0
	*Negative*	5	11	1	0	235	252
Pv/Pf Test	*P. falciparum*	26	0	12	0	2	40
	*P. vivax*	1	33	8	0	0	42
	*P. falciparum + P. vivax*	6	2	0	0	0	8
	*Negative*	7	17	1	0	235	260
Pf/Pan Test	*P. falciparum*	14	0	6	0	2	22
	*Pan*(non-*falciparum*)	0	33	5	0	0	38
	*P. falciparum + Pan*	18	5	9	0	1	33*
	*Negative*	8	14	1	0	234	257
	Total	40	52	21	0	237	350

* For the Pf/Pan device, these cases had both test lines visible, suggesting they either *P. falciparum* single infections or *P. falciparum*/non-*falciparum* mixed infections.

**Table 9:** Performance of different diagnosis methods with the nested PCR method as the gold standard

	**Microscopy (N=606)**	**Pf/Pan (N=606)**	**Microscopy (N=350)**	**Pv/Pf (N=350)**	**Pf/Pan (N=350)**
Sensitivity for *P. falciparum*	71.0 (67.0-74.9)	81.7 (78.4-85.1)	75.4 (70.4-80.4)	72.1 (66.9-77.3)	77.1 (72.2-81.9)
Sensitivity for *P. vivax*	73.3 (69.5-77.2)	64.6 (60.5-68.6)	74.0 (68.8-79.1)	58.9 (53.1-64.7)	63.5 (58.0-68.9)
Specificity	95.8 (93.9-97.6)	92.9 (90.5-95.3)	96.3 (93.9-98.7)	95.5 (92.9-98.1)	94.7 (92.0-97.5)

Data are presented as percentage (95% confidence interval; CI).

According to the microscopy results corrected by PCR, four and eight cases were false negative in 606 samples examined by the Pf/Pan test device for *P. falciparum* and *P. vivax*, respectively (Table 3). When mixed infections were excluded, three out of four *P. falciparum* cases had lower parasite density (<480 parasites/μL). Only one *P. falciparum* case with higher parasite density (8,800 parasites/μL) was not detected by the Pf/Pan test device, but was positive by the Pv/Pf test device. Five out of eight *P. vivax* cases were lower parasite density (<560 parasites/μL). The remaining three *P. vivax* cases with higher parasite density (1,880/μL, 2,760/μL and 15,840/μL) were not detected by the Pf/Pan test device. In the subset of 350 samples examined by the Pv/Pf test device (mixed infection not included, Table 5), two *P. falciparum* cases with lower parasite density (<480 parasites/μL) were false negative. Nine *P. vivax* cases were the false negative, of which three had lower parasite density (<320 parasites/μL), and three cases (parasite density of 1,320/μL, 2080/μL, 7,280/μL) were positive by the Pf/Pan test device. The other three cases with higher parasite density (1,880/μL, 2,760/μL and 15,840/μL) were not detected by both RDTs.

False positive and wrong species identification were observed with both RDTs. In all 606 samples (identified by microscopy and corrected by PCR, mixed infection not included) examined by the Pf/Pan test device, one *P. vivax* case, one *P. ovale* case, and eight negative cases showed the PfHRP2 line. Ten *P. vivax* cases showed not only the pan-pLDH line, but the PfHRP2 line. Seven negative cases showed both PfHRP2 and pan-pLDH lines (Table 7). In the subset of 350 samples analysed by the Pv/Pf test, two negative cases showed the PfHRP2 line. One *P. falciparum* case showed the Pv*-*pLDH line only. Two *P. vivax* cases showed not only the Pv-pLDH line, but the PfHRP2-line. Six *P. falciparum* cases (parasite density ranging from 240 to 34,400 parasites/μL) showed not only the PfHRP2 line, but also the Pv*-*pLDH line (Table 8). It has been reported that *P. falciparum* infections with high parasite densities may generate a false positive Pv-pLDH line [22-25]. For the Pv/Pf test devices, six out of 40 *P. falciparum* (corrected by PCR) with the Pv-pLDH line visible showed that cross reactions between different species occurred although parasite densities of some *P. falciparum* infections were not high. From these comparisons, the specificities of these devices need further improvement for areas with both *P. falciparum* and *P. vivax* malaria.

Since *P. falciparum* and *P. vivax* infections are treated with different drugs, it would be important to compare the different methods for detecting mixed species infections. PCR, microscopy and Pv/Pf test device detected 21, 11, and eight mixed-species infections in the 350 subset samples (Table 8), corresponding to 18.6%, 11.2% and 8.9% of positive cases detected by the respective methods.

## Discussion

RDTs are playing an essential role in the current malaria control/elimination campaign worldwide. In this study, the performance of two RDT devices commonly used at the China-Myanmar border area was evaluated. Compared to diagnosis by microscopy, both the Pf/Pan and Pv/Pf devices showed higher sensitivity (>87%) for detecting *P. falciparum* infections, whereas the sensitivity for detecting *P. vivax* infections was much lower (<74%). Both RDTs had similar levels of detection limits, and their performance was poor for infections with parasite densities below 500 parasites/μL for *P. falciparum* infections. Besides, since both devices rely on the PfHRP2 antigen for the detection of *P. falciparum*, *pfhrp2* gene deletions will lead to false-negative results [26]. A recent report showing up to 40% of *P. falciparum* parasites with *pfhrp2* deletions in South America [27] indicate that such RDTs would not perform well in these regions. It certainly warrants more detailed investigations in other malaria endemic regions.

When PCR was used as the gold standard, the detection sensitivity of both RDTs was reduced especially for the detection of *P. vivax* infections (<64%), although the specificity of the all detection methods remained above 92%. In addition, the sensitivity of conventional microscopy was also low, ranging from 71.0% to 77.4% for detecting both parasite species. This result is worrisome; since nearly 30% of the malaria cases was not correctly diagnosed by microscopy or RDTs and subsequently treated, these patients may constitute an important reservoir for transmission. Whereas low parasite density might be the most important limiting factor for microscopic diagnosis [6,28], the varying skills and experience of microscopists may also be responsible for the lower sensitivity of diagnosis [29]. In the past, the Pf/Pan test (Wondfo) has been evaluated for diagnosis of *P. falciparum* only and showed highly satisfactory result [30]. Recently, a CareStart™ kit (Pf/Pan) was evaluated in the nearby Yunnan province of China, which showed comparable results to microscopy for diagnosing *P. falciparum* and *P. vivax* infections [31]. Therefore, further evaluations of RDTs are needed to select better diagnostic tests with improved accuracy in this region.

Overall, both RDTs have good sensitivity for detecting *P. falciparum* infections, but the sensitivity for detecting *P. vivax* was much lower. This could be due to lower parasite density for *P. vivax* since this parasite selectively invades reticulocytes. However, comparison of the two RDTs showed that the sensitivities for *P. vivax* detection were not different between the high and low parasite densities (Table 6). This observation is difficult to explain and could be due to the lower number of infections analyzed in the <500 parasites/μL group. Furthermore, the arbitrary selection of 500 parasites/μL as the border line between high and low parasite densities may not be appropriate for *P. vivax*. In addition, the sensitivity to *P. vivax* may be affected by other factors. Therefore, better RDTs with significantly improved sensitivity for *P. vivax* are needed.

One important criterion for the selection of RDTs in the GMS is the ability to detect other human malaria species in addition to *P. falciparum*. The advantage of the Pf/Pan test is that it is designed to detect four human parasite species, which co-exist in the GMS. However, this device failed to detect the two *P. ovale* infections, suggesting that further improvement in sensitivity for detecting other malaria species is needed. It is also worth to note that the PCR method failed to identify any *P. knowlesi* infections, which is in stark contrast to the eaerlier report of some 30% of malaria cases in this region as mixed infections with *P. knowlesi*[16]. Therefore, the significantly lower prevalence of other malaria parasite species in this region and the generally low sensitivity of the Pf/Pan test for diagnosis of *P. ovale* and *P. malariae* suggest that such an advantage is hardly of any importance in this region. In comparison, the Pv/Pf test is specific for *P. falciparum* and *P. vivax,* which make up the majority of malaria infections in the GMS, although it would certainly miss detecting *P. malariae* and *P. ovale* infections. Because *P. falciparum* and *P. vivax* require different antimalarial treatments, correct diagnosis of mixed infections should be an important criterion for the selection of an RDT. Even though the Pv/Pf device is able to detect *P. falciparum* and *P. vivax* mixed infection, the detection rate was low and the eight mix infections detected by this device were all misdiagnosed when compared to the PCR result. This result may be due to reduced density of one parasite species in the presence of another due to competition in the same host. Consequently, with the current anti-malarial drug policy of this region, misdiagnosis of mixed species infections as single species infections would lead to improper drug treatments.

## Conclusions

The aim of this study was to evaluate the performance of two RDTs for malaria diagnosis at the China-Myanmar border area. PCR method was much more sensitive than microscopy and RDTs. Compared to microscopy, both RDTs demonstrated similar sensitivities for detecting *P. falciparum* infections but reduced sensitivity for detecting *P. vivax* infections. Neither of them had satisfactory results in detecting mixed species infections. Therefore, methods with improved sensitivity for diagnosing *P. vivax* malaria are needed. False negative results for the diagnosis of *P. falciparum* malaria call for further investigations on potential deletion of *pfhrp2* gene in this malaria-endemic region.

## Competing interests

The authors declare that they have no competing interests.

## Authors’ contributions

YJ, LN, WX, LP and ZZ carried out the experimental work and data analysis.WL, LS and ZG participated in data analysis. YJ performed manuscript writing. YC, QF and LC conceived the study and participated in the design of the study. All authors read and approved the final manuscript.
